# Effects of Bed Rest on Physical Performance in Athletes: A Systematic and Narrative Review

**DOI:** 10.1007/s40279-023-01889-y

**Published:** 2023-07-26

**Authors:** Barry A. Spiering, Jonathon Weakley, Iñigo Mujika

**Affiliations:** 1 Sports Research Laboratory, New Balance Athletics, Inc., Boston, MA USA; 2 School of Behavioural and Health Sciences, Australian Catholic University, McAuley at Banyo, Brisbane, QLD Australia; 3 Sports Performance, Recovery, Injury and New Technologies (SPRINT) Research Centre, Australian Catholic University, Brisbane, QLD Australia; 4 Carnegie Applied Rugby Research (CARR) Centre, Carnegie School of Sport, Leeds, UK; 5 Department of Physiology, Faculty of Medicine and Nursing, University of the Basque Country, Leioa, Basque Country Spain; 6 Exercise Science Laboratory, School of Kinesiology, Faculty of Medicine, Universidad Finis Terrae, Santiago, Chile

## Abstract

**Background:**

Athletes can face scenarios in which they are confined to bed rest (e.g., due to injury or illness). Existing research in otherwise healthy individuals indicates that those entering bed rest with the greatest physical performance level might experience the greatest performance decrements, which indirectly suggests that athletes might be more susceptible to the detrimental consequences of bed rest than general populations. Therefore, a comprehensive understanding of the effects of bed rest might help guide the medical care of athletes during and following bed rest.

**Objective:**

This systematic and narrative review aimed to (1) establish the evidence for the effects of bed rest on physical performance in athletes; (2) discuss potential countermeasures to offset these negative consequences; and (3) identify the time-course of recovery following bed rest to guide return-to-sport rehabilitation.

**Methods:**

This review was performed using the Preferred Reporting Items for Systematic Reviews and Meta-Analyses (PRISMA) guidelines. Four databases were searched (SPORTDiscus, Web of Science, Scopus, and MEDLINE/PubMed) in October of 2022, and studies were included if they were peer-reviewed investigations, written in English, and investigated the effects of horizontal bed rest on changes in physical capacities and qualities in athletes (defined as Tier 3–5 participants). The reporting quality of the research was assessed using a modified version of the Downs & Black checklist. Furthermore, findings from studies that involved participants in Tiers 1–2 were presented and synthesized using a narrative approach.

**Results:**

Our systematic review of the literature using a rigorous criterion of ‘athletes’ revealed zero scientific publications. Nevertheless, as a by-product of our search, seven studies were identified that involved apparently healthy individuals who performed specific exercise training prior to bed rest.

**Conclusions:**

Based on the limited evidence from studies involving non-athletes who were otherwise healthy prior to bed rest, we generally conclude that (1) bed rest rapidly (within 3 days) decreases upright endurance exercise performance, likely due to a rapid loss in plasma volume; whereas strength is reduced within 5 days, likely due to neural factors as well as muscle atrophy; (2) fluid/salt supplementation may be an effective countermeasure to protect against decrements in endurance performance during bed rest; while a broader array of potentially effective countermeasures exists, the efficacy of these countermeasures for previously exercise-trained individuals requires further study; and (3) athletes likely require at least 2–4 weeks of progressive rehabilitation following bed rest of ≤ 28 days, although the timeline of recovery might need to be extended depending on the underlying reason for bed rest (e.g., injury or illness). Despite these general conclusions from studies involving non-athletes, our primary conclusion is that substantial effort and research is still required to quantify the effects of bed rest on physical performance, identify effective countermeasures, and provide return-to-sport timelines in bona fide athletes.

**Trial Registration Number and Date of Registration:**

Registration ID: osf.io/d3aew; Date: October 24, 2022.

**Supplementary Information:**

The online version contains supplementary material available at 10.1007/s40279-023-01889-y.

## Key Points

**Table:** 

Our systematic review found zero scientific publications that investigated the effects of bed rest in bona fide athletes; therefore, greater scientific efforts should be made to elucidate the effects of bed rest in athletes.
As little as 3 days of bed rest can significantly impair upright endurance exercise performance; strength is likely impaired within 5 days of bed rest.
Combining multiple countermeasures that uniquely target the various underlying physiological changes that occur during bed rest can more effectively maintain physical performance, as opposed to relying on only one countermeasure used in isolation. Potentially effective countermeasures include fluid/salt loading, passive mechanical loading, protein supplementation, motor imagery training, passive blood flow restriction, electrical muscle stimulation, lower-body negative pressure, ‘anti-gravity suits’ that apply continuous resistance at the knee and ankle, and various pharmacological interventions.
Following bed rest of ≤ 28 days, we recommend at least 2–4 weeks of progressive rehabilitation (including strength training) to restore physical performance, although recovery timelines might need to be extended depending on the underlying reason for bed rest (e.g., illness or injury).

## Introduction

While pursuing the pinnacles of human performance, athletes inherently face injuries and illnesses. At their worst, these adversities can confine athletes to bed rest and potentially rob them of their hard-earned fitness. While existing review articles describe the effects of bed rest on physical performance in general populations [1–5], no existing review article specifically addresses athletes. This is concerning because previous research in otherwise healthy individuals indicates that those entering bed rest with the greatest physical performance level may experience the greatest performance decrements [1, 3, 6]. Specifically, individuals with a higher aerobic capacity prior to bed rest experience greater absolute losses in aerobic capacity following bed rest when compared with lesser-trained counterparts [3]. This finding indirectly suggests that athletes might be more susceptible to the detrimental consequences of bed rest than general populations. Moreover, the greatest rate of performance decline occurs during the earliest stages of bed rest [1–3]. For example, strength values follow a logarithmic decay in which there is rapid loss of strength within ~ 5 days of bed rest followed by a more gradual loss [2]. This evidence of potentially rapid and large performance decrements in athletes following bed rest further suggests that athletes might require longer periods of rehabilitation than lesser-trained individuals. In support of this notion, previous research [7] found that, following 21 days of bed rest, sedentary subjects required only ~ 7 to 10 days to return to their pre-bedrest aerobic performance values, whereas previously exercise-trained individuals required ~ 4 to 5 weeks to return to their substantially higher pre-bedrest values.

Without comprehensive insight into the available evidence, clinicians, scientists, and the sporting community may lack clear understanding of the consequences of bed rest specifically for athletes. Furthermore, recommendations for how to treat athletes during and following bed rest may be unclear. Therefore, we conducted a systematic review and provide a narrative discussion of the scientific literature to (1) establish the evidence for the effects of bed rest on physical performance in athletes; (2) identify potential countermeasures to offset bed rest-induced decrements in physical performance; and (3) provide timelines of recovery following bed rest, which could serve as the basis for evidence-based return-to-sport rehabilitation. Based on data from lesser-trained subjects, we hypothesized that (1) bed rest would rapidly (within ~ 3 days) degrade physical performance in athletes [8]; (2) many potentially effective countermeasures exist to protect physical performance during bed rest, and these countermeasures would be more effective when used in combination versus any one countermeasure used in isolation [1]; and (3) athletes require upwards of 4 weeks of rehabilitation following bed rest to fully recover their physical performance capabilities.

## Methods

### Search Strategy

Following Preferred Reporting Items for Systematic Reviews and Meta-Analyses guidelines [9], the academic databases SPORTDiscus, Web of Science, Scopus, and MEDLINE/PubMed were systematically searched in October of 2022 to identify English-language peer-reviewed original research studies that investigated the effects of horizontal bed rest on changes in physical capacities and qualities. Due to differences in database design, studies were identified by searching “abstracts, titles, and key words” in Scopus; “All Text” in SPORTDiscus and MEDLINE; and “All Fields” in Web of Science. The full search strategy for each database can be found in Electronic Supplementary Material 1 (ESM 1). Medical Subject Headings were not used when searching the MEDLINE/PubMed database. All search results were extracted and imported into a reference manager (Covidence, Veritas Health Innovation, Melbourne, Australia). A systematic review protocol that includes the review question, search strategy, exclusion criteria, and risk of bias assessment was registered on October 24, 2022, with the Open Science Framework (https://osf.io/d3aew).

### Selection Criteria

All duplicate studies were removed, and the titles and abstracts of all remaining studies were independently screened for relevance by two researchers (J.W. and B.S.). Studies that were deemed beyond the scope of the review were removed. Disagreements were resolved through discussion or via an additional researcher (I.M.). The full text of the remaining studies were then assessed for eligibility. To be eligible for inclusion, studies were required to (i) be original research investigations; (ii) be full-text articles written in English; (iii) be published in a peer-reviewed academic journal; (iv) be an investigation into apparently healthy adult humans between the ages of 18 and 50 years; (v) involve ‘bed rest’ that required participants to spend the entire length of the protocol in a horizontal position and did not involve decline or incline of the bed; (vi) provide objective evidence that details changes in physical performance or related physiological qualities (e.g., maximal oxygen consumption [*V*O_2max_]) from prior to bed rest to after the intervention; (vii) investigate outcomes in bona fide athletes who were classified as Tier 3–5 (i.e., highly trained/national level to world class [10]); and (viii) bed rest duration was ≥ 72 h. If it was deemed that a study did not meet the inclusion criteria, it was excluded from the analysis. The reference lists of all full-text screened studies were manually searched for any studies that were not retrieved in the initial search (i.e., ‘backwards searching’). Additionally, any articles that cited the full-text screened studies were searched (i.e., ‘forwards searching’). If a study was identified that might be eligible for inclusion it was subjected to the same assessment as previously described. Outcomes that were recorded were any objectively demonstrated changes in physical performance or relevant physiological qualities that occurred in response to bed rest. It should be noted that if during the search strategy a study was found that met all criteria except for inclusion point seven (i.e., participants were athletes that met the Tier 3–5 classification), this study was retrieved and noted. However, it was not included in the full search strategy outcomes. Instead, these data were used to narratively guide statements around changes in physical performance and physiological qualities following bed rest when data from bona fide athletes were unavailable.

### Assessment of Reporting Quality

The reporting quality of the research was assessed using a modified version of the Downs and Black checklist [11]. This method is valid for assessing the methodological reporting quality of intervention study designs and has previously been used by systematic reviews pertaining to sport science [12–15].

## Results

### Identification of Studies

**Fig. 1:**
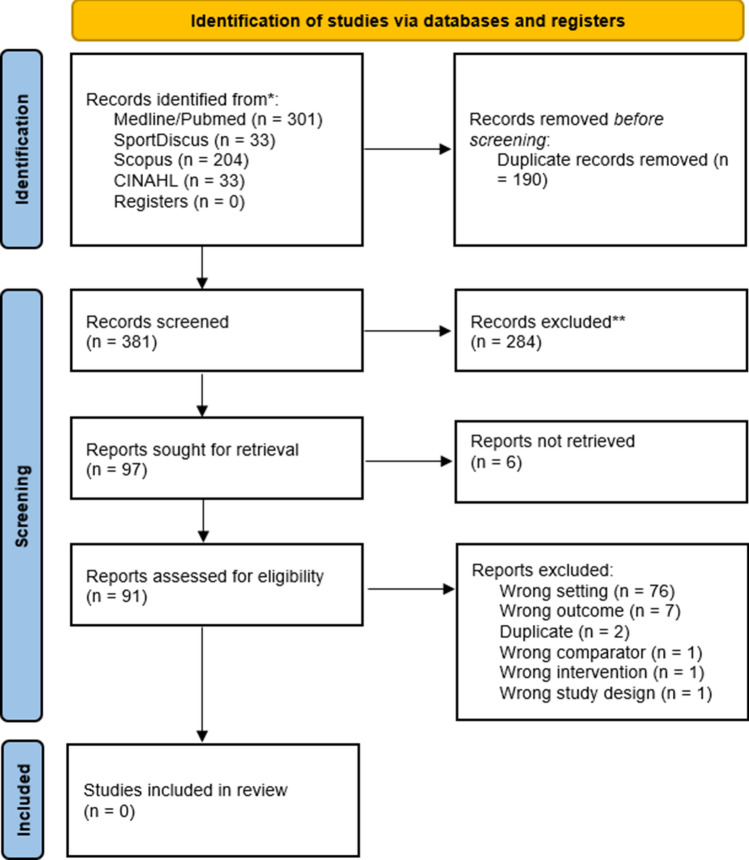
PRISMA flow diagram detailing the inclusion of papers throughout the search strategy. *Databases used in screening process; **Records removed following initial screening

The systematic search retrieved a total of 501 studies, with zero manuscripts found through screening of reference lists. Of these, 190 were removed as duplicates. The titles and abstracts of the remaining 381 studies were screened, with 97 manuscripts being sought for full-text screening. However, zero studies were identified that met the inclusion criteria. Because of this, questions 12–15 of the Preferred Reporting Items for Systematic Reviews and Meta-Analyses guidelines could not be completed. The identification process is outlined in Fig. 1.

### Research Reporting Quality

As no studies met the inclusion criteria, the methodological reporting quality could not be reported. However, seven studies that narratively guided the statements around changes in physical performance and physiological qualities following bed rest were assessed, with these found in ESM 2.

### Study Characteristics

No studies met the inclusion criteria of this review; this was largely attributed to no study including athletes who were in Tiers 3–5. Therefore, as no study provided evidence of changes in Tiers 3–5 level athletes following bed rest, we were unable to describe the changes in performance or physiological qualities. It should be noted that seven studies [8, 16–21] did follow a strict, horizontal bed rest procedure in apparently healthy, previously exercise-trained individuals (Tier 2: Trained/Developmental) while demonstrating changes in physical performance or relevant physiological qualities. If the study provided explicit evidence that subjects performed sport-specific training or competed locally, then Tier 2 status was assumed and the data were used to form the foundation of the discussion. Otherwise, without evidence of sport-specific training or local competitions, Tiers 0–1 (i.e., sedentary/recreationally active) status was assumed. Findings from Tiers 0–1 participants were only sparingly included in the discussion because these data likely have low generalizability to athletes [10].

## Discussion

Our systematic review identified zero studies that involved bona fide athletes confined to bed rest, which prevents us from making rigorous, evidence-based recommendations to the medical, scientific, and sporting communities. Nevertheless, we identified seven studies that involved regularly exercising subjects (Tier 2) [8, 16–21]. These studies serve as the basis for the discussion provided herein. Results from studies that involved sedentary or generally active individuals (Tiers 0–1) are only sparingly included in the discussion as a means to fill critical gaps when data from Tier 2 individuals did not exist. Importantly, due to the relatively lower-trained state of the individuals included in the discussion (Tiers 0–2), drawing direct conclusions relevant to higher-standard athletes (Tiers 3–5) is difficult [10]. Nevertheless, based on these studies that involved non-athletes, some general conclusions can be drawn. Specifically, bed rest rapidly (within 3 days) decreases upright endurance exercise performance [8], likely due to a concomitant loss in plasma volume [3]. Bed rest also decreases muscle strength within 5 days [2], likely due to neural factors as well as muscle atrophy [2, 22]. While confined to bed rest, combining multiple countermeasures that specifically target the various underlying physiological changes might help maintain physical performance. For example, fluid/salt supplementation can offset reductions in aerobic capacity by maintaining plasma volume [18–21], whereas dietary supplementation (i.e., increased protein) [23] and passive therapies (e.g., passive mechanical loading [24] or electrical muscle stimulation [23]) might help maintain muscle performance (e.g., strength). Finally, the timeline of recovery in performance following bed rest likely depends on the duration of bed rest, the initial performance levels of the individual, and the underlying reason for bed rest (e.g., illness or injury) [3].

### Research Reporting Quality of the Studies Involving Tier 2 Participants

Although no studies met the criteria of our systematic review, we identified seven studies that involved regularly exercising subjects (Tier 2) (ESM 2) [8, 16–21]. The reporting quality of these seven studies was assessed using a modified version of the Downs and Black checklist [11]. All seven studies had a reporting quality score of at least six out of a maximum possible score of nine. Items that were consistently not achieved included question 3 (inclusion/exclusion criteria of participants, *n* = 5 studies), question 10 (actual probability values reported for main outcomes, *n* = 6 studies), and question 18 (adequate description of appropriate statistical analyses, *n* = 3 studies). To improve the quality of future research, authors should report all inclusion/exclusion criteria for participants, all statistical tests, and the actual probability values of main outcomes.

### Effects of Bed Rest on Physical Performance

While no studies were found that objectively demonstrated changes in physical performance in athletes (Tiers 3–5), seven studies described the effects of bed rest on endurance-related outcome variables in regularly exercising (Tier 2) individuals (see Table 1). These studies found that endurance exercise performance, *V*O_2max_, and lactate threshold declined after just 3 days of bed rest. Moreover, Smorawiński et al. [8] found that the magnitude of decrement in *V*O_2max_ correlated positively with the initial values (i.e., participants with greater initial *V*O_2max_ experienced the greatest loss), and that endurance-trained subjects had significantly larger decrements in *V*O_2max_ and lactate threshold than sedentary subjects. Collectively, these findings support the notion that athletes might be more susceptible to the negative consequences of bed rest than their lesser-trained counterparts.

**Table 1:** Effects of bed rest on physical performance-related outcomes in previously exercise-trained individuals (results are organized in ascending order according to the duration of bed rest)

Study	Subjects	Duration	Exercise test	Outcome (% change in the group mean)
Smorawiński et al. [8]	Tier 2 (ET) men (*n* = 10)	3 days	Upright	MIEL ↓ (− 14.3%) *V*O_2_max ↓↓ (− 16.5%) LT ↓↓ (− 24.8%)
Smorawiński et al. [8]	Tier 2 (ST) men (*n* = 10)	3 days	Upright	MIEL ↓ (− 10.0%) *V*O_2_max ↓ (− 10.4%) LT ↔ (− 10.1%)
Zorbas et al. [18–21]^a^	Tier 2 (ET) men (*n* = 40)	7 days	Supine	*V*O_2_max ↓ (~ − 3.9%) PV ↓ (~ − 12.3%) FFM ? (~ − 1.1%)
Zorbas et al. [19–21]^a^	Tier 2 (ET) men (*n* = 30)	15 days	Supine	VO_2_max ↓ (~ − 4.7%) PV ↓ (~ − 9.0%) FFM ? (~ − 1.9%)
Balsam and Leppo [16]	Tier 2 (ET) men (*n* = 7)	17 days	Unknown body position	*V*O_2_max ↓ (− 19.5%)
Sketch et al. [17]	Tier 2 (TS) men (*n* = 8)	18 days	Upright	MIED ↓ (− 8.1%) *V*O_2_max ↓ (− 9.2%)
Zorbas et al. [19–21]^a^	Tier 2 (ET) men (*n* = 30)	30 days	Supine	*V*O_2_max ↓ (~ − 8.4%) PV ↓ (~ − 18.2%) FFM ↔ (~ − 2.4%)

*ET* previously endurance-trained individuals, *FFM* fat free mass, *LT* lactate threshold (defined as the workload [Watts] concomitant with a rapid increase in blood lactate), *MIED* maximal incremental exercise duration (time), *MIEL* maximal incremental exercise load (Watts), *PV* plasma volume, *ST* previously strength-trained individuals, *TS* individuals previously involved in recreational team sports, *VO*_*2*_ oxygen consumption, ~ indicates approximate change when averaged across multiple studies by the same lead author, ? indicates inconsistent findings (some studies showing non-significant changes and other studies showing significant decreases), ↔ indicates non-significant change, ↓ indicates significant decrease, ↓↓ indicates significant decrease that was also significantly greater than the corresponding value for ST

^a^All studies by Zorbas et al. [16–19] were exceptionally homogeneous in terms of study design, study participants, and results; therefore, the results were averaged across studies and reported as approximate overall means

Regarding the time-course of the decay in endurance performance and associated physiological outcomes, the available data in Tier 2 individuals prove difficult to interpret. Smorawiński et al. [8] and Sketch et al. [17] utilized upright exercise tests, whereas Zorbas et al. [18–21] utilized supine exercise tests, and Balsam and Leppo [16] did not report whether upright or supine exercise was used. However, two general conclusions emerge from these studies (as summarized in Table 1). First, *V*O_2max_ rapidly decays during the initial 1–2 weeks of best rest; subsequently, the rate of decay decreases. According to Lee et al. [3], a mechanistic explanation for this finding is that the rapid, initial decline in *V*O_2max_ is closely mirrored by a concomitant decrease in plasma volume, thus reducing cardiac output during exercise. Thereafter, the slower decay in *V*O_2max_ is due to a combination of cardiovascular structural changes as well as muscular changes (e.g., reduced oxidative enzymes). Second, bed rest more profoundly affects upright exercise than supine exercise. The underlying mechanism is that, following bed rest, upright exercise elevates heart rate and decreases stroke volume to a greater extent than supine exercise, due to the effects of gravity on body fluid distribution [3].

With regards to the effects of bed rest on muscle-specific outcomes, Zorbas et al. [18–21] found inconsistent changes in fat-free mass (sometimes non-significant and sometimes significant declines) in endurance-trained individuals following 7–30 days of bed rest. One possible explanation for the inconsistent findings is that the individual studies by Zorbas et al. [18–21] may have lacked sufficient statistical power to detect changes in muscle mass. However, as demonstrated by Marusic et al. [2] (involving primarily Tier 0–1 individuals), clear and significant reductions in muscle mass do occur following bed rest. According to Nunes et al. [23], a mechanistic explanation for reduced muscle mass during bed rest includes disuse-induced reductions in muscle protein synthesis, both while in a fasted state (as normally encountered during an overnight fast, for example), as well as following feeding. Finally, it should be noted that of the seven studies that involved bed rest in previously exercise-trained individuals, none investigated muscle performance metrics such as strength. Nevertheless, in Tier 0–1 individuals, bed rest reduces muscle strength within 5 days [2]. Therefore, countermeasures to offset bed-rest induced decrements in muscle performance are warranted (see Sect. 4.2).

### Potential Countermeasures to Offset Bed Rest-Induced Decrements in Physical Performance

Exercise potently protects physical performance during bed rest [3, 23, 25, 26]. Nevertheless, when athletes are confined to bed rest due to injury or illness, exercise countermeasures might not always be feasible. Therefore, research has investigated the efficacy of non-exercise countermeasures to protect physical performance when confined to bed rest [1, 23, 24, 27–29]. With respect to Tier 2 individuals, the only studies examining non-exercise countermeasures all involved fluid/salt supplementation [18–21]. In these studies, the daily administered dose of fluid/salt supplementation was 26–30 mL water per kg body mass and 0.1 g sodium chloride per kg body mass. Fluid/salt supplementation completely preserved (and, surprisingly, significantly increased) supine *V*O_2max_ values during bed rest, likely because fluid/salt supplementation also maintained plasma volume [19–21]. Of note, fluid/salt supplementation was provided prior to bed rest (for 7–15 days), as well as during the bed rest period. Whether fluid/salt supplementation effectively maintains *V*O_2max_ when solely administered during the bed rest period remains to be determined. Furthermore, all existing studies that describe the effects of fluid/salt supplementation in Tier 2 individuals originated from the same laboratory [19–21]. Therefore, further research with differing cohorts is needed to corroborate these findings.

Studies that involved Tier 0–1 individuals have evaluated a broader array of potential non-exercise countermeasures besides fluid/salt loading. For example, passive mechanical loading [24], protein supplementation [23], motor imagery training [28], passive blood flow restriction (BFR) [27], electrical muscle stimulation [23], lower-body negative pressure (LBNP) [1, 29], ‘anti-gravity suits’ that apply continuous resistance at the knee and ankle [1], and various pharmacological interventions [1, 30] might have merits for protecting physical performance during bed rest. However, the extent to which these non-exercise countermeasures protect performance in previously exercise-trained individuals (Tier 2 and beyond) requires further research. Nevertheless, we briefly review these non-exercise countermeasures below.

Several countermeasures exist to help maintain muscle strength during bed rest. For example, some countermeasures protect muscle strength by imparting mechanical tension within the muscle, which stimulates muscle protein synthesis [23, 31]. Passive mechanical loading (i.e., 2.5 h of continuous passive motion using a machine, performed four times per day for an overall total of 10 h of loading) [24], electrical muscle stimulation (i.e., 30–45 min per session performed during three to five sessions per week) [23, 31], and ‘anti-gravity suits’ (for 10 consecutive hours per day) [1] all impart mechanical tension within the muscle and all demonstrate some effectiveness for preserving muscle size and strength during bed rest. Similarly, protein ingestion (e.g., 16.5 g essential amino acids plus 30 g carbohydrate given 3 times per day [32]; or 0.06 g of leucine per kg of body mass per meal [33]) stimulates muscle protein synthesis, thus supporting dietary protein supplementation as another non-exercise countermeasure [23]. Alternatively, some research has also found that inducing intramuscular metabolic stress via passive BFR (5 sets of 5 min of occlusion performed on 2 sessions per day) can preserve muscle mass and strength during immobilization [27]. While loss of muscle mass during bed rest typically coincides with loss of muscle strength [2], changes in neural factors also contribute to strength loss [22]. Mentally performing resistance exercise (also known as ‘motor imagery training’) involves similar neural activation as actually performing resistance exercise [34]. As such, a large body of evidence demonstrates that motor imagery training (mentally replicating strenuous resistance exercise sessions, performed during multiple sessions per week) can improve strength in otherwise healthy individuals [34], as well as help preserve strength during immobilization [35]. Therefore, motor imagery training represents an intriguing countermeasure for preserving muscle strength during bed rest. Collectively, this research indicates that creating muscle mechanical tension (via passive mechanical loading, electrical muscle stimulation, or ‘anti-gravity suits’), providing supplemental dietary protein, and creating intramuscular metabolic stress (via passive BFR) might help maintain muscle strength during bed rest via preserving muscle mass, whereas motor imagery training might help maintain muscle strength via neural mechanisms.

Preserving orthostatic tolerance might be another key for maintaining physical performance during bed rest. When standing, gravity pulls body fluids toward the lower extremities; however, during bed rest, the horizontal body position causes body fluids to redistribute more centrally [36]. The prolonged reduction in orthostatic stress during bed rest commonly results in orthostatic hypotension when the individual eventually returns to an upright posture (i.e., when bed rest ends) [1]. However, utilizing LBNP during bed rest simulates the effects of gravity and causes associated orthostatic stress; consequently, LBNP used during bed rest (one or more daily sessions of ~ 15 min at approximately − 30 to − 50 mmHg) lessens the orthostatic hypotension that occurs when the individual returns to an upright posture [1]. Importantly, many studies that investigated LBNP as a countermeasure involved head-down bed rest [1]; therefore, the effectiveness of LBNP in horizontal bed rest in athletes requires additional research. Nevertheless, daily LBNP exposure during bed rest might help preserve orthostatic tolerance, and thus upright exercise performance, in athletes.

Pharmacological agents represent another classification of non-exercise countermeasures for athletes confined to bed rest. For example, in addition to LBNP, pharmacological agents might also protect orthostatic tolerance during bed rest. Pharmacological interventions previously used to maintain orthostatic tolerance during bed rest include atropine, propranolol, clonidine, ephedrine, indomethacin, fludrocortisone, and midodrine [1]. Given the various mechanisms of action of these pharmacological agents, research has also explored whether some combination of agents (e.g., combination of fludrocortisone, dextroamphetamine, and atropine) might be most effective in managing orthostatic tolerance [37]. However, due to individual responsiveness, it seems that the ideal agent or combination of agents for maintaining orthostatic tolerance has not been identified [1, 37]. Besides orthostatic tolerance, the effectiveness of pharmacological agents (e.g., testosterone) for preserving muscle mass and strength has also been investigated. In general, testosterone maintains lean body mass but does not appear to help maintain muscle strength [38]. Importantly, though, athletic doping laws must be considered when using pharmacological agents (e.g., testosterone, ephedrine, atropine) to maintain physical performance during bed rest.

Importantly, it has been strongly recommended [1, 3] to combine several countermeasures while an individual is confined to bed rest, as opposed to relying on only one countermeasure used in isolation. Given that bed rest affects multiple physiological systems [39], and given that each physiological system affects physical performance in a unique way, then perhaps combining multiple countermeasures to simultaneously offset bed rest-induced plasma volume losses (e.g., fluid/salt supplementation), orthostatic hypotension during upright exercise (e.g., LBNP), muscle atrophy (e.g., protein supplementation, passive mechanical loading, passive blood flow restriction, electrical muscle simulation, and/or ‘anti-gravity suits’), and deficits in neural drive to muscle (e.g., motor imagery training) could all be used to more fully maintain physiological attributes and thus physical performance. Therefore, clinicians and scientists should consider combining multiple countermeasures to protect performance during bed rest.

### Recovery of Physical Performance Following Bed Rest

Of the seven studies that investigated the effects of bed rest in previously exercise-trained individuals (Tier 2), none described the time-course of recovery following bed rest. This represents a critical gap in our collective understanding of return-to-sport timelines for athletes following bed rest. A review by Lee et al. [3] (relying on data from Tier 0–1 individuals) concluded that recovery of endurance capacity following bed rest is related to the duration of bed rest as well as pre-bedrest fitness levels. Specifically, the authors [3] indicated that short durations of bed rest (approximately 2 weeks) require approximately 1 week of recovery, but longer durations of bed rest might require 2–4 weeks for full recovery. Lee et al. [3] further concluded that those with higher initial endurance levels need more time to recover, indicating that the general guidance in the previous sentence might not fully translate to endurance-trained individuals (e.g., ≥ Tier 2).

Regarding neuromuscular performance, Brooks et al. [40] found that 14 days of re-ambulation plus resistance exercise was sufficient to completely recover muscle strength (but not muscle size) following 28 days of best rest. However, 14 days of re-ambulation alone (without resistance exercise) was insufficient to restore muscle strength. Similarly, Pišot et al. [41] found that 14 days of re-ambulation plus rehabilitation that included resistance exercise was sufficient to restore muscle size and strength following 14 days of bed rest. Conversely, Abe et al. [42] and Berg et al. [43] found that re-ambulation alone effectively restored strength in Tier 0–1 individuals following 3–5 weeks of bed rest. Collectively, we conservatively recommend approximately 2–4 weeks of progressive rehabilitation (including strength training [44]) to restore muscle strength, as well as endurance exercise, following bed rest of ≤ 28 days. It is worth re-emphasizing that these bed rest-induced decrements in performance in Tier 0–1 individuals might be smaller than the decrements experienced by higher-performing athletes. Moreover, the recovery timelines described herein are based on otherwise healthy individuals who are voluntarily confined to bed rest for research purposes, not ill or injured individuals who are confined to bed rest for medical reasons. Therefore, higher-performing athletes who are confined to bed rest for medical reasons might require longer rehabilitation to fully restore physical performance following bed rest.

### Limitations

Two primary limitations underlie the conclusions drawn within this review. First, due to the absence of scientific evidence describing the consequences of bed rest in bona fide athletes, we were forced to draw our conclusions from studies that involved previously exercise-trained (Tier 2) individuals and, in some cases, from recreationally active (Tier 1) or even sedentary (Tier 0) individuals. Indirect evidence suggests that athletes might be more susceptible to the negative consequences of bed rest [1, 3], potentially obscuring the general conclusions drawn herein. Second, the studies included in this review all involved subjects who were otherwise healthy. While this experimental evidence in healthy individuals is essential for rigorously testing hypotheses, the generalizability of this information for athletes confined to bed rest for underlying illness or injury must be considered. These two limitations ultimately mean that the scientific community currently has very little understanding of the impact of illness-induced or injury-induced bed rest in bona fide athletes. Nevertheless, these two limitations provide an important foundation for guiding future research.

### Future Directions

Of foremost importance for guiding the care of athletes confined to bed rest, future research must include bona fide athletes. The studies included in the present review involved otherwise healthy individuals confined to bed rest. While such experiments are critical for describing the physical and physiological consequences of bed rest and evaluating various countermeasures, the likelihood of otherwise healthy athletes voluntarily consenting to such experiments seems low, at best. Therefore, alternative research designs might be more successful. For example, research that involves Tier 3–5 athletes who are (unfortunately) confined to bed rest for medical reasons would likely yield important insights and fill key gaps in our collective understanding. To further guide the design of such research, the following paragraphs provide specific recommendations for consideration.

Future research should measure physical performance as soon as medically feasible following bed rest, and then compare that information to the athlete’s pre-bedrest physical testing and training records. Additionally, the precise performance metrics should be matched to the athlete’s sport (e.g., measuring *V*O_2max_, lactate threshold, running economy, for distance runners or, alternatively, measuring muscle size, strength, power in Olympic weightlifters) [45]. Such information could provide important insights on the magnitude and time-course of performance decrements in bona fide athletes. Similarly, future research should examine the rate of recovery in performance throughout rehabilitation. Such information is critical for creating evidence-based return-to-sport programs to rehabilitate athletes following bed rest. In addition to studying physical performance decrements (e.g., endurance and strength), future research should also examine the physiological underpinnings. For example, the initial loss of strength in general populations confined to bed rest exceeds the loss in muscle mass, suggesting that neural factors (e.g., supraspinal drive) as well as muscular factors independent of atrophy (e.g., single fiber excitability and mechanical properties, as well as architectural factors) strongly contribute to the initial loss in strength [2, 46, 47]. Over longer periods of bed rest, however, a large fraction (~ 79%) of the strength loss during bed rest can be attributed to losses in muscle mass [2]. This mechanistic information provides the foundation for future countermeasures that specifically target the underlying physiological mechanism.

Finally, future research should report any interventions used during bed rest (e.g., diet, medications, countermeasures), as well as interventions used during recovery (e.g., rehabilitation programs, dietary supplementation). For example, in addition to being an effective countermeasure during bed rest, protein supplementation might also accelerate recovery and rehabilitation [48]. Furthermore, an innovative approach to implementing countermeasures during and following bed rest would be to simultaneously combine several countermeasures, as this approach might more effectively maintain/improve physical performance than any one countermeasure used in isolation. As an example, muscle contractions (naturally occurring or artificially induced via electrical stimulation) sensitize muscles to the anabolic effects of protein supplementation on muscle protein synthesis; therefore, simultaneously combining muscle contractions with protein supplementation may expedite the recovery of muscle mass [48].

## Conclusions

**Fig. 2:**
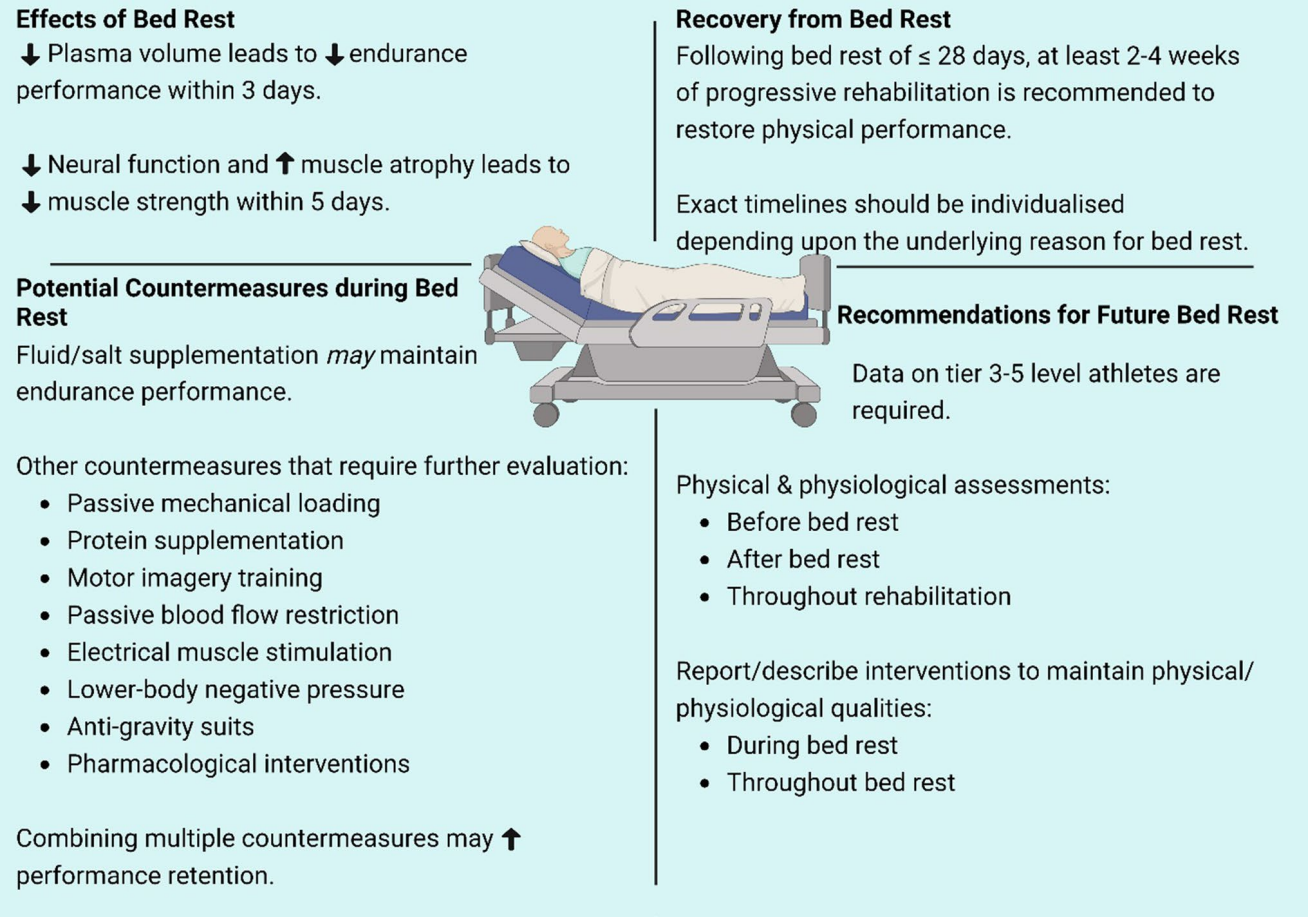
Summary of the effects of bed rest on physical performance in athletes

Our systematic review of the literature using a rigorous criterion of ‘athletes’ (Tiers 3–5) found zero studies. Nevertheless, seven studies investigated the effects of bed rest in previously exercise-trained individuals (Tier 2), and we sparingly relied on studies that involved sedentary or generally active individuals (Tiers 0–1) to reach general conclusions (Fig. 2). These studies found that bed rest rapidly (within 3 days) decreases upright endurance exercise performance, likely due to a rapid loss in plasma volume. Insufficient data from trained individuals (Tier 2) is available to detail the consequences of bed rest on muscle performance (e.g., strength); nevertheless, data from Tier 0–1 individuals indicates a reduction in muscle strength within 5 days of bed rest. To offset the detrimental consequences of bed rest, combining multiple non-exercise countermeasures likely maximizes the overall effectiveness, as opposed to choosing only one countermeasure used in isolation. Following bed rest of ≤ 28 days, we recommend at least 2–4 weeks of progressive rehabilitation (including strength training) to restore physical performance, although recovery timelines might need to be extended depending on the underlying reason for bed rest (e.g., illness or injury). Overall, these conclusions drawn from Tier 0–2 individuals provide only limited insight into the consequences of bed rest for Tier 3–5 athletes. Therefore, our primary conclusion is that substantial work is still needed to quantify the effects of bed rest on physical performance, identify effective countermeasures, and provide return-to-sport timelines using bona fide athletes.

### Practical Recommendations

The findings from this review paper support several evidence-based recommendations for practitioners who care for athletes during and following bed rest. During bed rest, the medical staff must consider the underlying reason for bed rest (e.g., injury or illness) as well as the corresponding interventions necessary to treat the injury/illness (e.g., medication, surgery). After thorough consideration of these factors, the medical staff then can choose from an array of potentially effective countermeasures, including fluid/salt loading, passive mechanical loading, protein supplementation, motor imagery training, passive blood flow restriction (BFR), electrical muscle stimulation, lower-body negative pressure (LBNP), and ‘anti-gravity suits’ that apply continuous resistance. We generally recommend prioritizing interventions that have a high likelihood of being effective combined with a low likelihood of producing further harm (e.g., following musculoskeletal injury, motor imagery training presents a high likelihood for preserving muscle strength combined with a low likelihood of exacerbating the underlying injury). In addition, we generally recommend against using pharmacological interventions intended solely for preserving physical performance due to the potential for doping/ethical violations. Following bed rest, the sports medicine and strength and conditioning staff should collaboratively design a rehabilitation program of appropriate quality, quantity, and duration. In general, following bed rest of ≤ 28 days, we recommend at least 2–4 weeks of progressive rehabilitation (including strength training) to restore physical performance. Clearly, though, recovery timelines might need to be extended depending on the underlying reason for bed rest (e.g., illness or injury).

### Supplementary Information

Below is the link to the electronic supplementary material.

Supplementary file1 (DOCX 17 KB)

## Declarations

### Availability of data and material

All data and material reported in this systematic review are from peer-reviewed publications.

### Registration

This systematic review was registered with the Open Science Framework (registration ID: osf.io/d3aew).
