# Supplementary material for: Effects of Bed Rest on Physical Performance in Athletes: A Systematic and Narrative Review
Source: Sports Med. 2023 Jul 26;53(11):2135–46. doi: 10.1007/s40279-023-01889-y (PMC10587175; doi:10.1007/s40279-023-01889-y)
Supplement: Supplementary file 1 — Supplementary file1 (DOCX 17 KB) [file 40279_2023_1889_MOESM1_ESM.docx]

**Journal**

Sports Medicine

**Title**

Effects of Bed Rest on Physical Performance in Athletes: A Systematic and Narrative Review

**Authors**

Barry A. Spiering^1^, Jonathon Weakley^2,3,4^, Iñigo Mujika^5,6^

**Affiliations**

^1^Sports Research Laboratory, New Balance Athletics, Inc., Boston, MA, USA

^2^School of Behavioural and Health Sciences, Australian Catholic University, Brisbane, QLD, Australia

^3^Sports Performance, Recovery, Injury and New Technologies (SPRINT) Research Centre, Australian Catholic University, Brisbane, QLD, Australia

^4^Carnegie Applied Rugby Research (CARR) Centre, Carnegie School of Sport, Leeds, United Kingdom

^5^Department of Physiology, Faculty of Medicine and Nursing, University of the Basque Country, Leioa, Basque Country

^6^Exercise Science Laboratory, School of Kinesiology, Faculty of Medicine, Universidad Finis Terrae, Santiago, Chile

**Supplementary Material 1**

Scopus

TITLE-ABS-KEY ( trained OR athlete OR fit OR healthy AND "bed rest" OR bedrest OR bed-rest AND strength OR power OR "anaerobic endurance" OR "aerobic capacity" OR endurance OR fitness OR "lean body mass" OR "muscle mass" OR "Sprint speed" OR speed OR "oxidative capacity" OR "ventilatory threshold" OR "Aerobic power" OR "Respiratory exchange ratio" OR "stroke volume" OR "Carbohydrate oxidation" OR "fat oxidation" OR "Maximal oxygen consumption" OR "running economy" OR "cycling economy" OR "aerobic performance" OR "anaerobic performance" OR "anaerobic threshold" AND NOT mice OR disease OR injur* OR "back pain" OR stroke OR animal OR review OR pregnancy OR "older adults" )

Medline/Pubmed

(((Trained OR athlete OR Fit OR Healthy) AND ("bed rest" OR bedrest OR bed-rest)) AND (strength OR power OR "anaerobic endurance" OR "aerobic capacity" OR Endurance OR fitness OR "lean body mass" OR "muscle mass" OR "Sprint speed" OR speed OR "oxidative capacity" OR "ventilatory threshold" OR "Aerobic power" OR "Respiratory exchange ratio" OR "stroke volume" OR "Carbohydrate oxidation" OR "fat oxidation" OR "Maximal oxygen consumption" OR "running economy" OR "cycling economy" OR "aerobic performance" OR "anaerobic performance" OR "anaerobic threshold")) NOT (mice OR rat OR disease OR Injur* OR "back pain" OR stroke OR animal OR review OR pregnancy OR "older adults")

SportDiscus and CINAHL

( Trained OR athlete OR Fit OR Healthy ) AND ( "bed rest" OR bedrest OR bed-rest ) AND ( strength OR power OR “anaerobic endurance” OR “aerobic capacity” OR Endurance OR fitness OR "lean body mass" OR "muscle mass" OR "Sprint speed" OR speed OR “oxidative capacity” OR “ventilatory threshold” OR “Aerobic power” OR “Respiratory exchange ratio” OR “stroke volume” OR “Carbohydrate oxidation” OR “fat oxidation” OR “Maximal oxygen consumption” OR “running economy” OR “cycling economy” OR “aerobic performance” OR “anaerobic performance” OR “anaerobic threshold” ) NOT ( mice OR disease OR Injur* OR "back pain" OR stroke OR animal OR review OR pregnancy OR “older adults” )

**Supplementary Material 2.** Methodological reporting quality of studies involving Tier 2 participants, which were used in the narrative review. Studies were assessed using modified Downs & Black checklist.

| Reference | Reporting | | | | | | Internal Validity | | | |
| --- | --- | --- | --- | --- | --- | --- | --- | --- | --- | --- |
|  | 1 | 2 | 3 | 6 | 7 | 10 | 16 | 18 | 20 | Total |
| Balsam et al. [16] | 1 | 0 | 1 | 1 | 1 | 1 | 1 | 0 | 1 | 7 |
| Sketch et al. [17] | 1 | 1 | 1 | 1 | 1 | 0 | 1 | 0 | 1 | 7 |
| Smorawiński et al. [8] | 1 | 1 | 0 | 1 | 1 | 0 | 1 | 0 | 1 | 6 |
| Zorbas et al. [18] | 1 | 1 | 0 | 1 | 1 | 0 | 1 | 1 | 1 | 7 |
| Zorbas et al. [19] | 1 | 1 | 0 | 1 | 1 | 0 | 1 | 1 | 1 | 7 |
| Zorbas et al. [20] | 1 | 1 | 0 | 1 | 1 | 0 | 1 | 1 | 1 | 7 |
| Zorbas et al. [21] | 1 | 1 | 0 | 1 | 1 | 0 | 1 | 1 | 1 | 7 |
